# Do Diagnostic Nerve Blocks Affect the Starting Dose of Botulinum Neurotoxin Type A for Spasticity? A Case-Control Study

**DOI:** 10.3390/toxins16090388

**Published:** 2024-09-06

**Authors:** Mirko Filippetti, Stefano Tamburin, Rita Di Censo, Roberto Aldegheri, Elisa Mantovani, Stefania Spina, Marco Battaglia, Alessio Baricich, Andrea Santamato, Nicola Smania, Alessandro Picelli

**Affiliations:** 1 Department of Neurosciences, Biomedicine and Movement Sciences, University of Verona, 37100 Verona, Italy; mirko.filippetti@univr.it (M.F.); rita.dicenso@univr.it (R.D.C.); roberto.aldegheri@studenti.univr.it (R.A.); elisa.mantovani@univr.it (E.M.); nicola.smania@univr.it (N.S.); alessandro.picelli@univr.it (A.P.); 2 Canadian Advances in Neuro-Orthopedics for Spasticity Consortium (CANOSC), Kingston, ON K7K 1Z6, Canada; 3 Neurorehabilitation Unit, Department of Neurosciences, University Hospital of Verona, 37100 Verona, Italy; 4 Spasticity and Movement Disorders ‘ReSTaRt’ Unit, Physical Medicine and Rehabilitation Section, Policlinico Riuniti Hospital University of Foggia, 71122 Foggia, Italy; spinastefania.ss@gmail.com (S.S.); andrea.santamato@unifg.it (A.S.); 5 Physical and Rehabilitative Medicine, Department of Health Sciences, University of Eastern Piedmont “A. Avogadro”, 28100 Novara, Italy; marco.battaglia@uniupo.it; 6 Department of Biomedical Sciences, Humanitas University, Via Rita Levi Montalcini 4, Pieve Emanuele, 20090 Milan, Italy; alessio.baricich@hunimed.eu; 7 IRCCS Humanitas Research Hospital, Via Manzoni 56, Rozzano, 20089 Milan, Italy

**Keywords:** botulinum neurotoxin, drug utilization, muscle spasticity, nerve block, ultrasonography

## Abstract

One of the aims of diagnostic nerve blocks is to identify the overactive muscles that lead to a specific spasticity pattern. However, to date, there is no evidence on how nerve blocks may affect botulinum neurotoxin-A (BoNT-A) dose in patients with spasticity. This case-control study aims to assess the role of diagnostic nerve block in defining BoNT-A starting dose at first treatment. Patients with upper and lower limb spasticity treated for the first time with BoNT-A were retrospectively divided into two groups: Group 1 (n = 43) was evaluated with clinical assessment and diagnostic nerve block; Group 2 (n = 56) underwent clinical assessment only. Group 1 was injected with higher BoNT-A doses in some muscles (i.e., flexor digitorum profundus, soleus), and received a higher BoNT-A cumulative dose with a larger number of injected muscles for some spasticity patterns (i.e., “clenched fist”, “flexed fingers”, “adducted thigh”). Diagnostic nerve block may help the clinician to optimize and personalize the BoNT-A dose since the first BoNT-A treatment.

## 1. Introduction

Spasticity is a positive symptom of the upper motor neuron syndrome, which may impair motor function and quality of life [1,2,3]. Timely detection and targeted treatment play pivotal roles in appropriately managing spasticity [4]. Diagnostic nerve blocks (DNBs) have emerged as a widely used procedure for managing spasticity in order to provide some key information for differential diagnosis and treatment planning [5,6].

Diagnostic nerve blocks are helpful in the evaluation and treatment of patients with complex spasticity secondary to neurological diseases. In particular, DNBs (a) may help to differentiate between spastic muscle overactivity and contracture, (b) offer a transient clinical and functional change, upon which the effects of botulinum neurotoxin injection, neurolysis or selective neurectomy may be inferred before treatment and (c) better define the therapeutic objectives in accordance with the patient’s preferences [5,6,7,8,9,10,11,12].

Botulinum toxin type A (BoNT-A) is a first-line treatment for upper and lower limb spasticity that effectively reduces muscle overactivity [13,14,15,16]. The accuracy of BoNT-A administration (i.e., the injection of adequate doses into specific muscles, which underlie different focal spasticity patterns) is crucial in order to achieve the desired outcomes. Among different injection techniques for BoNT-A, ultrasound has gained a growing diffusion because it enhances the precision and safety of the procedure [17,18]. This is due to the real-time visualization of the target muscles, the surrounding anatomical structures as well as the needle and the injected BoNT-A [19].

It is common clinical practice to start BoNT-A treatment of focal spasticity at low doses and then titrate upwards to optimize the effect on subsequent BoNT-A injections. However, there is concern that this approach may lead to the injection of an insufficient or inadequate BoNT-A dose, which may lead to the loss of response and treatment drop-out [20,21,22,23]. Furthermore, personalized allocation of BoNT-A doses to the needs of single patients with spasticity may be complex because target muscles are often injected according to the labeled doses and the choice of muscles that usually sustain spasticity patterns, and not considering specific features that may vary from patient to patient. DNBs may help address these issues in that they allow us to infer the effect of BoNT-A treatment in patients with spasticity [6,7,8,9,10,11,12]. In that respect, DNB was found to provide greater reduction in spastic muscle overactivity than labeled doses of BoNT-A in adult patients with lower limb post-stroke spasticity [24]. On the other hand, the impact of DNBs on the choice of the dose of BoNT-A administered at first injection, and the BoNT-A distribution across different muscles, has yet to be studied, to date.

The aim of this study is to investigate the role of DNBs in optimizing the starting dose (i.e., the total dose administered at first injection) of BoNT-A in patients with spasticity at their first treatment. These findings may contribute to defining BoNT-A treatment protocols according to a personalized and tailored perspective.

## 2. Results

This case-control study retrospectively included 99 adult patients affected by upper and/or lower limb spasticity who received BoNT-A injection for the first time. They were divided into two groups according to the methods described below. Detailed clinical and demographic characteristics of the patients are reported in Table 1. No significant differences were observed between groups regarding demographic, clinical and BoNT-A treatment features.

Table 2 and Table 3 provide, separately for Group 1 and Group 2, the number of subjects who had each muscle treated and the mean dose of BoNT-A injected in the muscle for the upper (Table 2) and lower limb (Table 3), respectively.

For the upper limb, a statistically significant difference in the mean BoNT-A injected dose was found between groups only for the flexor digitorum profundus muscle (Group 1: 66.7 ± 24.5 U, Group 2: 45.2 ± 13.5 U; *p* = 0.02; Table 2).

For the lower limb, a statistically significant difference in the mean BoNT-A injected dose was found between groups only for the soleus muscle (Group 1: 102.6 ± 24.1 U, Group 2: 87.2 ± 17.9 U; *p* = 0.03; Table 3).

When considering the spasticity patterns, a statistically significant difference was found between groups in the mean BoNT-A injected dose (i.e., higher dose in Group 1) and the number of treated muscles (i.e., higher number in Group 1) for the clenched fist and flexed fingers within upper limb patterns and the adducted thigh within lower limb patterns (Table 4) [26,27,28].

## 3. Discussion

We found no significant difference between the two groups regarding the total BoNT-A dose administered or the number of muscles treated. However, a statistically significant difference was observed in the mean BoNT-A dose administered to the flexor digitorum profundus muscle in upper limb spasticity, in that the dose was significantly larger in patients who underwent clinical evaluation combined with DNB than those whose injection was based on clinical assessment only. Similarly, in patients with lower limb spasticity, the soleus muscle received a higher BoNT-A dose when the injection was guided by clinical evaluation and DNB than clinical evaluation alone.

For the flexor digitorum profundus, we found that the average starting dose in the clinically based treatment group (i.e., 45 U) was lower than the average starting dose suggested by WE MOVE™ 3.0 [29] (i.e., 80 U). In contrast, the mean dose in the clinical and DNB-based treatment group (i.e., 67 U) was slightly lower than recommended. Of interest in our study is a larger number of patients who underwent the flexor digitorum superficialis injection, with higher BoNT-A doses than the flexor digitorum profundus. We may speculate that DNB of the flexor digitorum profundus and superficialis may lead to more tailored treatment of these muscles, depending on the spasticity features of each patient, and this may explain the higher flexor digitorum profundus starting dose in Group 1.

The soleus muscle showed similar findings, as patients who underwent clinical assessment alone (Group 2) had a lower starting dose (i.e., 87 U) compared to those evaluated by clinical evaluation combined with DNB (Group 1), where the BoNT-A dose (i.e., 103 U) was comparable to the one suggested by WE MOVE™ 3.0 (i.e., 100 U) [29]. This discrepancy was not observed for the gastrocnemius muscles, where no statistically significant difference between the two groups was found. We speculate that a DNB for the soleus motor branch may help not only in the evaluation of clinical outcomes of spasticity (e.g., modified Ashworth scale, Tardieu scale, passive range of motion), but also to verify the stability of the tibial pendulum during the second rocker in the stance walking phase, thus leading to a higher starting dose in Group 1. Conversely, the inability to predict the effect of the administered dose based solely on clinical evaluation may justify the lower starting dose and the less frequent treatment in Group 2. The greater starting dose for the soleus muscle in Group 1 may be due to its prominent role in post-stroke spasticity patients, who represent nearly half of the two samples.

Notably, the average starting dose for patients receiving clinically based treatment (Group 2) was sometimes higher than the average starting dose recommended by WE MOVE™ 3.0 [29] or both the upper and lower limb spasticity. In our study, the BoNT-A treatment was conducted in a hub center by expert clinicians, which could explain this higher starting dose. This finding aligns with the results reported by Esquenazi et al. [30], who reported that the administered BoNT-A doses statistically differed according to the physician’s experience, with more experienced physicians administering higher BoNT-A doses in both the upper and lower limb muscles than less experienced ones. This point might have affected our study’s ability to detect significant differences between the two groups for muscles other than the FDP and soleus. Furthermore, this may have hampered the identification of significant differences when conducting a spasticity pattern-based analysis, especially when only one or two muscles per pattern (e.g., in the upper limb “adducted shoulder”, “flexed elbow”, “flexed wrist”, “thumb in palm” and lower limb “flexed knee”, “extended knee”, “flexed toes” patterns) were treated, as discussed below.

Analyzing data according to the spasticity patterns of upper and lower limb spasticity [26,28], we found a statistically significant difference (i.e., higher dose and higher number of treated muscles in Group 1) for the “clenched fist” and the “flexed fingers” patterns in the upper limb and the “adducted thigh” pattern in the lower limb.

The “clenched fist” and “flexed fingers” patterns involve the flexor digitorum profundus and superficialis and the flexor pollicis longus muscle, and the statistical difference for these patterns between groups might be related to the higher dose of BoNT-A injected in the flexor digitorum profundus. It should be noted that the significance of one pattern drives the other, as there is a consistent overlap of muscles across these two patterns.

The “adducted thigh” pattern is ascribed to hyperactivity of the adductor longus, gracilis, iliopsoas and medial hamstring muscles. In our Group 1 patients, the gracilis and adductor longus muscles were the most frequently injected, while the medial hamstrings were more frequently injected in Group 2 patients. We may speculate that the different BoNT-A injection types in the two groups might have contributed to the significant difference for this spasticity pattern. In our clinical practice, DNBs for assessing the “adducted thigh” pattern prioritize the obturator anterior over the medial hamstring branches due to the satisfactory clinical outcomes of blockade of the former. Conversely, the medial hamstrings might be overtreated in the clinically based group. Still, we cannot draw definitive conclusions on this issue since no clinical data were analyzed in the present study, besides BoNT-A dose and number of injected muscles.

Surprisingly, we found no significant difference between the two groups in the “equinovarus foot pattern”, despite it involving the soleus muscle, whose dose was significantly higher in Group 1. This discrepancy might depend on the preferential involvement of other muscles (i.e., gastrocnemius medialis, gastrocnemius lateralis, flexor digitorum longus, flexor hallucis longus, tibialis posterior) in our sample.

Previous studies support the role of DNB as a valuable screening tool in deciding whether to treat spasticity and in guiding and mediating the treatment goals of patients [12,30]. This study adds some information to this topic, as it supports the role of DNBs in guiding the starting dose towards a more targeted and effective distribution of BoNT-A across injected muscles. Dose optimization may reduce the need to go beyond the labeled doses and contribute to achieve better treatment goals [12]. From this perspective, DNB might help clinicians tailor the treatment, given that optimal BoNT-A doses may vary from patient to patient and that approximately 60% of physicians would use higher doses if there were no label restrictions [31].

The findings from this study contribute to the existing knowledge on the beneficial role of DNB in spasticity management, potentially encouraging broader adoption of this technique in neurorehabilitation settings. One of the possible clinical implications of this new evidence might be to increase the confidence of clinicians to inject higher starting BoNT-A doses within the labeled range for each muscle to achieve the optimal outcome rapidly.

Future prospective studies should explore not only the starting dose and the faster and more effective attainment of therapeutic goals but also offer a more comprehensive evaluation of all treatment cycles during follow-up, as well as additional clinical outcomes to fully understand the long-term implications and benefits of DNB-guided BoNT-A treatment.

This study has some limitations: (a) we focused on BoNT-A dose without spasticity outcome data, (b) causes of spasticity varied across patients and etiology was not analyzed as a covariate, (c) the retrospective design of the study resulted in the selection of patients who had complete clinical records, and this may have resulted in the exclusion of some patients.

## 4. Conclusions

This is the first study investigating the relationship between DNB and the BoNT-A starting dose. Our findings support the role of DNB in optimizing the BoNT-A starting dose in patients with upper and lower limb spasticity, giving a more targeted and effective distribution of the dose compared to a treatment plan based only on clinical evaluation. Although the present data cannot confirm this hypothesis, which will require future studies, we speculate that DNBs might also improve the optimal distribution of BoNT-A dose in the follow-up of patients with spasticity.

## 5. Materials and Methods

### 5.1. Study Design

A retrospective evaluation was conducted on the medical records of patients who underwent their initial assessment for spasticity at the dedicated outpatient clinic of the University Hospital of Verona between January 2020 and April 2024. The inclusion criteria for the study required (a) patients to be over 18 years of age, (b) to have a diagnosis of disabling spasticity affecting the upper and/or lower limbs, (c) to be referred for their first assessment for BoNT-A injection for spasticity (i.e., no previous BoNT-A injection) and (d) to have or have not received DNB as part of their spasticity assessment/management. Patients were only included if it was possible to obtain all data from their clinical records for the purposes of the study. Patients enrolled in the study were categorized into two groups according to their treatment approach. Group 1 comprised patients who received their initial treatment plan based on clinical findings combined with DNB procedures, while Group 2 included patients whose treatment was determined clinically without using DNB procedures. Data collected from the clinical records included the patient’s age, the cause of spasticity, the nerves/muscles evaluated by DNB (for Group 1 only), the date of the first BoNT-A treatment, the type of BoNT-A used, the specific muscles treated, the dose administered to each muscle and the total starting dose of BoNT-A used. In order to limit the variability resulting from different operators, all DNB procedures and BoNT-A treatments were done by the same two clinicians, who are experts in the management of spasticity patients.

### 5.2. DNB Procedures

In Group 1, all patients underwent DNB to guide the treatment of spasticity. As part of our standard care, DNBs were performed using a 22-gauge, 80 mm, ultrasound-faceted tip echogenic needle designed for nerve blocks (SonoPlex STIM, Pajunk, Geisingen, Germany). The target nerves, whether primary nerve trunk or muscular branches, were identified using both ultrasound guidance (MyLab 70 XVision system, Esaote SpA, Genoa, Italy; linear probe set at 13 MHz) and electrical nerve stimulation (Plexygon, Vygon, Padua, Italy). Upon identification of the target nerve, indicated by an appropriate muscle response to electrical stimuli (1 Hz, 100 μs, 0.5 mA), lidocaine 2% was administered. In accordance with the French clinical guidelines for peripheral motor nerve blocks in the physical and rehabilitation medicine setting, the maximum dose of lidocaine administered per DNB session was 2 mg/kg [5]. For the upper limb, potential DNB targets included the lateral and medial pectoral nerves, the musculocutaneous nerve and the median and ulnar nerves. For the lower limb, possible targets encompassed the anterior obturator nerve, the femoral nerve and its motor branches to the rectus femoris, vastus medialis and vastus lateralis muscles and the tibial nerve main trunk along with its motor branches [6,32,33,34,35,36].

### 5.3. BoNT-A Treatment

All patients received BoNT-A treatment. To exclude a possible pharmacodynamical interaction between lidocaine and BoNT-A, according to our usual care, BoNT-A was injected in a separate session than DNB. Group 1 underwent the treatment based on clinical evidence combined with diagnostic nerve blocks (DNBs). For Group 2, the selection of muscles for injection was based only on clinical evaluation [26,27,28]. BoNT-A injections were guided by ultrasound (MyLab 70 XVision system, Esaote SpA, Genoa, Italy; linear probe set at 13 MHz) and administered using a 22-gauge, 40 mm needle. Injection sites were identified in accordance with established protocols [17,18]. The dilution ratios were 100 U in 2 mL for Onabotulinumtoxin-A and Incobotulinumtoxin-A, and 500 U in 2.5 mL for Abobotulinumtoxin-A. The dose, number of injection sites and total dose per session adhered to the label recommendations for each type of BoNT-A.

### 5.4. Data Analysis

Statistical analysis was conducted using the SPSS software package version 21.0 (SPSS, Chicago, IL, USA). The normality of distribution for continuous variables was assessed using the Shapiro–Wilk test. For continuous variables, an independent samples *t*-test was used in the case of normal distribution, while the non-parametric Mann–Whitney U test was applied when the distribution was not normal. The chi-squared (χ^2^) test was applied to categorical variables. The significance threshold was set at *p* < 0.05 (two-tailed) for all the tests.

To make the doses comparable across different BoNT-A types, a 1:3 ratio was used between Onabotulinumtoxin-A and Incobotulinumtoxin-A, with respect to Abobotulinumtoxin-A, as outlined by Scaglione et al. [25].

Pattern-specific analysis was performed by grouping muscles based on previously published guidelines for each pattern. For upper limb patterns, the muscle groups were classified as proposed by Simpson et al. [26], and for lower limb patterns, the classification by Esquenazi et al. [28] was applied. The upper limb patterns and involved muscles were as follows [26]: adducted shoulder (pectoralis major), flexed elbow (brachioradialis, biceps brachii, brachialis), clenched fist (flexor digitorum superficialis, flexor digitorum profundus, flexor pollicis longus, lumbricalis), flexed wrist (flexor carpi radialis, flexor carpi ulnaris), flexed fingers (flexor digitorum profundus, flexor digitorum superficialis), thumb in palm (flexor pollicis longus, opponens pollicis). For the lower limbs, patterns and involved muscles were as follows [28]: adducted thigh (adductor longus, gracilis, iliopsoas, medial hamstrings), flexed knee (gracilis), extended knee (rectus femoris), equinovarus foot (gastrocnemius medialis, gastrocnemius lateralis, soleus, flexor digitorum longus, flexor hallucis longus, tibialis posterior), flexed toes (flexor digitorum longus, flexor digitorum brevis, flexor hallucis longus).

## Figures and Tables

**Table 1:** Demographic, clinical and BoNT-A treatment features of patients.

	Group 1 (DNB + Clinical Assessment, n = 43)	Group 2 (Clinical Assessment Only, n = 56)	*p*
Demographic			
Age, year	56.5 ± 16.9	56.7 ± 14.5	0.95
Male %	62.8	58.9	0.70
Clinical			
Cause of spasticity			0.38
Ischemic stroke, %	34.9	39.3	
Hemorrhagic stroke, %	9.3	5.4	
Multiple sclerosis, %	20.9	23.2	
CP, %	18.6	5.4	
SCI, %	0.0	8.9	
TBI, %	7.0	5.4	
Other, %	9.3	12.5	
BoNT-A treatment			0.40
Onabotulinumtoxin-A, %	32.6	30.4	
Incobotulinumtoxin-A, %	25.6	16.1	
Abobotulinumtoxin-A, %	41.8	53.5	
Total BoNT-A dose, U *	245.5 ± 153.1	279.7 ± 137.6	0.25
Upper limb BoNT-A dose, U *	155.1 ± 82.0	201.0 ± 102.4	0.07
Lower limb BoNT-A dose, U *	229.6 ± 118.5	224.0 ± 109.6	0.84
Injected muscles, number	3.8 ± 1.9	4.1 ± 2.4	0.51
Muscle injected upper limb, number	2.9 ± 1.2	3.3 ± 1.6	0.29
Muscle injected lower limb, number	3.4 ± 1.7	3.0 ± 1.6	0.40

Legend. CP: cerebral palsy; SCI: spinal cord injury; TBI: traumatic brain injury; BoNT-A: botulinum neurotoxin-A; U: unit. * Onabotulinum toxin-A and Incobotulinum toxin-A to Abobotulinum toxin-A ratio: 1:3 [25]. Statistical significance set at *p* < 0.05.

**Table 2:** Number of subjects who had each upper limb muscle treated and mean starting dose for each muscle.

Muscle	Group 1 (DNB + Clinical Assessment)		Group 2 (Clinical Assessment Only)		*p* Dose
	n	Dose	n	Dose	
Pectoralis major	9	79.6 ± 23.2	6	75.0 ± 27.4	0.74
Biceps brachii	8	95.8 ± 45.2	5	80.0 ± 32.0	0.48
Brachialis	10	73.3 ± 19.6	7	79.8 ± 10.6	0.40
Brachioradialis	0	-	4	58.3 ± 20.4	-
Pronator teres	0	-	2	50.0 ± 0.0	-
Flexor carpi radialis	8	52.1 ± 20.8	10	50.0 ± 16.7	0.82
Flexor carpi ulnaris	8	43.7 ± 8.6	8	51.9 ± 16.7	0.25
Flexor digitorum profundus	13	66.7 ± 24.5	7	45.2 ± 13.5	0.02 *
Flexor digitorum superficialis	22	71.6 ± 38.2	13	57.7 ± 17.5	0.15
Flexor pollicis longus	14	42.5 ± 22.0	5	31.7 ± 10.9	0.18
Opponens	5	20.3 ± 6.8	3	19.4 ± 4.8	0.84
Lumbricalis	2	45.0 ± 7.1	3	40.0 ± 20.0	0.72

Legend. DNB: diagnostic nerve block. * Statistically significant comparison (*p* < 0.05).

**Table 3:** Number of subjects who had each lower limb muscle treated and mean starting dose for each muscle.

Muscle	Group 1 (DNB + Clinical Assessment)		Group 2 (Clinical Assessment Only)		*p*
	n	Dose	n	Dose	
Gracilis	6	63.9 ± 45.2	6	56.9 ± 20.0	0.74
Adductor longus	5	83.3 ± 37.3	2	41.7 ± 0.0	0.07
Ileopsoas	1	100.0 ± 0.0	1	100.0 ± 0.0	-
Medial hamstrings	0	-	7	76.1 ± 8.8	-
Rectus femoris	13	89.1 ± 14.6	3	91.7 ± 14.4	0.80
Gastrocnemius medialis	21	71.8 ± 18.7	21	76.6 ± 17.6	0.40
Gastrocnemius lateralis	23	73.2 ± 18.8	23	79.71 ± 18.6	0.24
Soleus	26	102.6 ± 24.1	13	87.2 ± 17.9	0.03 *
Tibialis posterior	13	71.1 ± 11.1	5	68.3 ± 17.1	0.74
Flexor digitorum longus	10	59.2 ± 51.9	2	62.5 ± 17.7	0.88
Flexor hallucis longus	10	40.8 ± 13.9	2	50.0 ± 0.0	0.07
Flexor digitorum brevis	0	-	5	14.3 ± 6.2	-
Extensor hallucis longus	2	50.0 ± 0.0	1	50.0 ± 0.0	-

Legend. DNB: diagnostic nerve block. * Statistically significant comparison (*p* < 0.05).

**Table 4:** Number of muscles and mean starting dose of BoNT-A for each upper and lower limb spasticity pattern.

Pattern	Group 1 (DNB + Clinical Assessment)			Group 2 (Clinical Assessment Only)			*p* Dose	*p* Muscles
	n	Dose	Muscles	n	Dose	Muscles		
Adducted shoulder	9	79.6 ± 23.2	1.0 ± 0.0	6	75.0 ± 27.4	1.0 ± 0.0	0.74	-
Flexed elbow	15	100.1 ± 48.3	1.3 ± 0.6	12	99.3 ± 43.5	1.3 ± 0.6	0.97	0.79
Clenched fist	22	142.7 ± 77.7	2.5 ± 0.7	16	80.2 ± 35.7	1.8 ± 0.7	0.01 *	0.01 *
Flexed wrist	10	85.2 ± 24.2	1.8 ± 0.4	9	91.5 ± 40.8	1.8 ± 0.4	0.69	0.91
Flexed fingers	22	110.9 ± 56.7	1.7 ± 0.5	15	71.1 ± 27.4	1.4 ± 0.5	0.01 *	0.07
Thumb in palm	18	38.7 ± 25.0	1.1 ± 0.2	7	31.0 ± 14.2	1.1 ± 0.4	0.34	0.59
Adducted thigh	7	128.4 ± 82.7	1.7 ± 0.5	14	75.5 ± 15.1	1.2 ± 0.4	0.03 *	0.04 *
Flexed knee	6	63.9 ± 45.2	1.0 ± 0.0	6	56.9 ± 20.0	1.0 ± 0.0	0.74	-
Extended knee	13	89.1 ± 14.6	1.00 ± 0.0	3	91.7 ± 14.4	1.0 ± 0.0	0.80	-
Equinovarus foot	39	202.2 ± 104.5	2.7 ± 1.3	25	205.7 ± 62.3	2.6 ± 0.7	0.87	0.84
Flexed toes	10	86.9 ± 29.1	2.0 ± 0.0	5	55.2 ± 43.6	1.4 ± 0.5	0.19	0.07

Legend. DNB: diagnostic nerve block. * Statistically significant comparison (*p* < 0.05).

## Data Availability

Original data are available by contacting the corresponding authors upon reasonable request.
